# Physiotherapeutic Interventions for Individuals Suffering From Plantar Fasciitis: A Systematic Review

**DOI:** 10.7759/cureus.42740

**Published:** 2023-07-31

**Authors:** Manali A Boob, Pratik Phansopkar, Kamya J Somaiya

**Affiliations:** 1 Department of Musculoskeletal Physiotherapy, Ravi Nair Physiotherapy College, Datta Meghe Institute of Higher Education & Research, Wardha, IND

**Keywords:** therapeutic intervention, foot orthosis, strengthening, stretching, intrinsic muscle, plantar fasciitis

## Abstract

The foot and the lower leg comprise the ankle joint complex. The foot is crucial for the maintenance of posture. Frequently, overuse or repeated microtrauma to the fascia causes plantar fasciitis. This review aims to suggest the efficacy of various plantar fasciitis (PF) interventions based on modifications in clinical results. This review included studies from 2019 to March 2023 identified through a systematic literature search. The measures used to predict improvement in pain, discomfort, and foot function symptoms included the Visual Analog scale, Numerical Pain Rating Scale, Pressure Point Threshold by algometer, Weight-Bearing Lunge Test by inch tape, and range of motion. The review included 20 studies that fulfilled the inclusion criteria. Therapeutic interventions included insoles, foot orthosis, foam roller stretching, manual stretching, muscle strengthening, intrinsic muscle activities, extracorporeal shock wave lithotripsy, dry needling, laser, ultrasound, and others, which resulted in pain reduction, improved foot function, and ease of everyday routine. All therapeutic strategies used impacts resulting from minimal to maximal recovery. Various advanced approaches are more effective than conventional physical therapy. In conclusion, conservative therapeutic strategies with manual techniques, orthoses, and alternative intervention strategies can be combined to effectively relieve pain and improve function and overall results. Further high-quality studies are essential to learn more about the ideal dose, treatment approaches, and long-term impacts of these therapies.

## Introduction and background

The foot plays a crucial role in postural control and locomotion, and it has been described that foot pathologies have a potentially adverse effect on an individual’s quality of life [1]. The 26-foot bones, tibia, and fibula make up the foot and ankle joint. Talocalcaneal (subtalar), tibiotalar (talocrural), and transverse-tarsal joints comprise the ankle joint complex [2]. Locomotion and other daily activities depend heavily on the ankle joint complex, which creates the kinetic connection that allows the lower leg to interact with the ground [2]. Foot movements have been frequently reported to result in the following three fundamental planes: sagittal, frontal, and transverse [3]. The arch of the foot includes the following three domes: one transverse dome and two longitudinal domes. The ligaments and tendons in the foot support these arches created by the tarsal and metatarsal bones [4]. About 11-15% of all foot problems are significantly contributed by plantar fasciitis, leading to frequent foot discomfort. Regular microtrauma or severe stress to the fascia are the usual causes of plantar fasciitis [5]. This condition causes extensive discomfort and stiffness in the feet. The band that connects the calcaneus to the toes on the bottom of the foot is called the plantar fascia. Persistent standing, overweight, excess foot pronation, jogging, and poor ankle dorsiflexion are some risk factors that damage the plantar fascia [6]. Plantar fasciitis can be treated with various physiotherapy approaches, including calf, hamstring, and Achilles tendon stretching; strengthening of the foot’s intrinsic muscles; myofascial release; and manual techniques. Therefore, the primary goal of this review is to analyze and evaluate whether traditional interventional strategies and newly launched modalities can benefit individuals suffering from plantar fasciitis. The objective of this review is to compare and analyze the effects of conservative physiotherapeutic intervention and advanced rehabilitation in patients suffering from plantar fasciitis in randomized and non-randomized clinical trials.

Pathophysiology of plantar fasciitis

Traditionally, it was believed that plantar fasciitis is induced by mechanical damage in which the plantar fascia was subjected to excessive tensile strain, which caused tiny tears and persistent inflammation. According to current theories, plantar fasciitis develops through a deterioration of fascia, which is why it is sometimes referred to as *fasciosis *rather than *fasciitis*, where maximum stress is the main factor in the etiology [7]. Gap junctions between fibrocytes specifically perceive the increased fascial stress, which subsequently mediates alterations in the extracellular matrix, leading to myxoid deterioration and breakage of the plantar fascia [8].

Clinical manifestations of plantar fasciitis

Most patients have severe, stabbing, and burning pain that starts in the posteromedial region of the calcaneus and gradually spreads to the medial dome of the foot. The pain is typically worse in the early morning hours or following a period of inactivity, with the initial few footsteps being the most uncomfortable and gradually improving as the individual walks [9]. Additionally, chronic overuse issues such as jogging might cause pain. In many patients, a clinical finding showed tenderness around the medial heel or medial arch. Anatomical anomalies, including deviations from normal arches, restrictions in the mobility of the ankle joint, scar tissue, and thinning of the plantar fat pad, should be examined during a foot examination [10].

Diagnostic investigations

The primary criteria of diagnosis rely on the history and clinical assessment. Individuals who feel discomfort and pain while assessing the heel area and those who experience discomfort and soreness while touching their heels, taking their first steps in the morning, or after continuous sitting, which exerts pressure on the foot. The proximal plantar fascia experiences excruciating discomfort when the great toe is passively dorsiflexed. Diagnostic imaging is rarely necessary. Only patients with associated heel pathologies should undergo magnetic resonance imaging (MRI) or ultrasonography. Observations of enlarged fascia thicknesses and aberrant tissue on MRI or ultrasonography confirm plantar fasciitis [11].

## Review

Study selection

**Figure 1:**
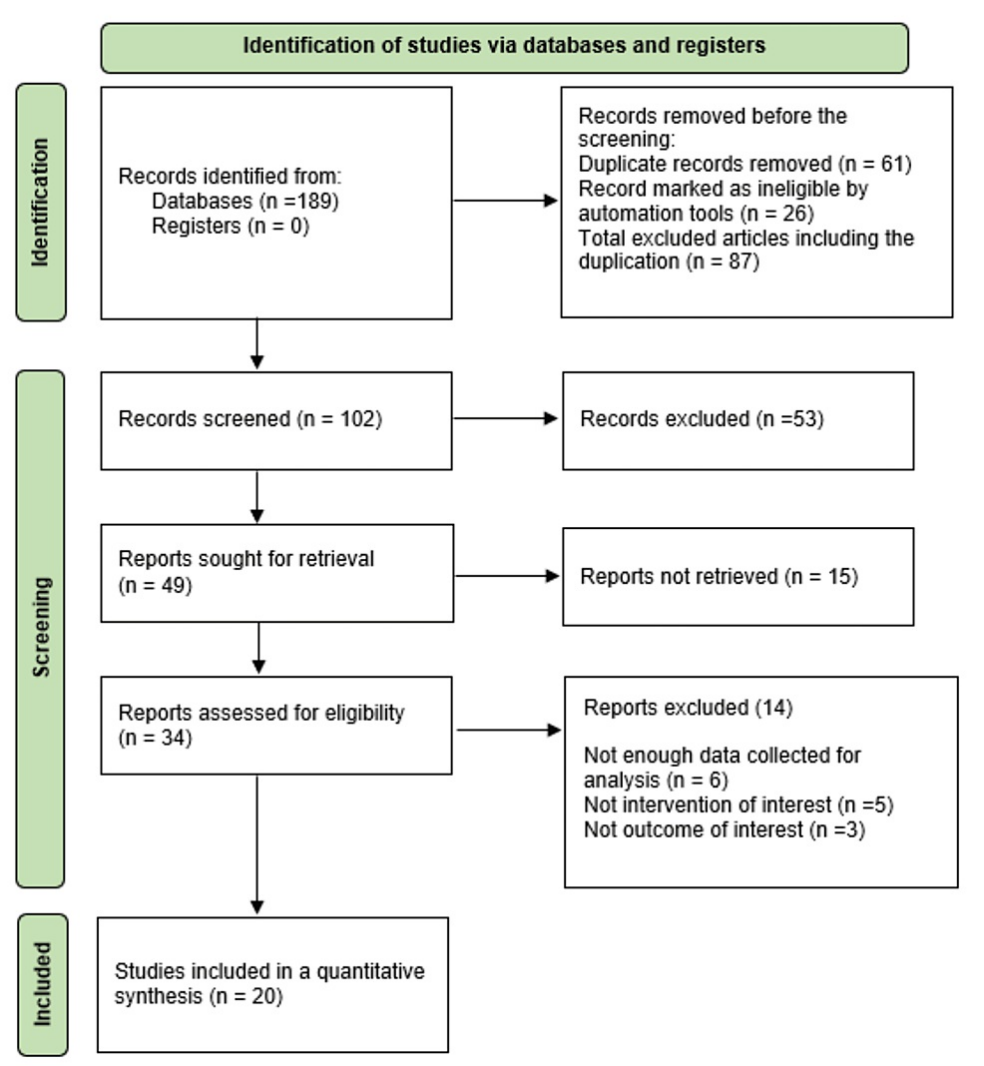
Preferred Reporting Items for Systematic Reviews and Meta-Analyses flowchart.

The English-language literature was searched on Google Scholar and PubMed for randomized and non-randomized clinical trials to evaluate the impact of various physiotherapeutic interventions on restoring plantar fasciitis symptoms in patients with plantar fasciitis. Younger and older adults suffering from heel pain or pain that aggravates on the first step in the morning and having a positive windlass test were included in the study. A total of 189 articles were searched from 2019 to March 2023 using a combination of keywords plantar fasciitis or plantar fasciopathy, intrinsic foot exercises and stretching, and advanced physiotherapy. Out of the 189 articles, 20 were eligible for full-text review. The result of selecting articles is illustrated in Figure 1, which shows a search of the database and data extraction. Table 1 presents a summary of the articles that were reviewed for plantar fasciitis.

**Table 1:** A summary of the articles reviewed for plantar fasciitis. VAS: Visual Analog Scale; MMT: manual muscle testing; ROM: range of motion; IASTM: instrument-assisted soft tissue mobilization; BMI: body mass index; NPRS: Numerical Pain Rating Scale; FFI: Foot Functional Index; WBLT: Weight-Bearing Lunge Test; FADI: Foot and Ankle Disability Index; PPT: pain pressure thresholds; HILT: high-intensity laser therapy; LLLT: low-level laser therapy; ESWT: extracorporeal shock wave therapy; KT: kineso-tapping; AOFAS: American Orthopedic Foot and Ankle Society; IRT: infrared thermometer; FAAM: Foot and Ankle Ability Measures Scale; FHSQ: Foot Health Status Questionnaire; CFO: custom foot orthotics

Authors and year of publication	Study type (sample Size)	Outcome measure	Intervention	Results	Conclusions
Nadeem et al. 2023 [5]	A randomized control trial (64 individuals with plantar fasciitis)	VAS, MMT, ROM	Group A: conventional treatment combined with the IASTM approach utilizing an Ergon tool. Group B: conventional treatment	All individuals in the interventional group had a significant enhancement (p = 0.05) in their outcomes. The dorsiflexion showed no appreciable improvement. Intragroup finding revealed improvement in dorsiflexion ranges in both groups (p = 0.05)	Five weeks of IASTM with the Ergon technique is a beneficial intervention in relieving pain and enhancing strength and ankle joint mobility
Takroni et al. 2022 [12]	A cross-sectional online survey study (93 participants with plantar fasciitis)	BMI, activity level and frequency of exercise, and NPRS	Foot insole/heel cushion orthosis	There was a substantial reduction in the pain level score after wearing foot orthosis	Foot orthotics are one of the most effective interventions for plantar fasciitis
Shabbir et al. 2022 [13]	A comparative study (56 individuals with plantar fasciitis)	FFI	Group A: movement combined with mobilization, overnight splint, routine, and physiotherapy. Group B: routine physiotherapy	From baseline to the third week, group A showed significant improvement than group B	Both groups improved considerably, but group A improved considerably more than group B
Manzoor and Munir 2022 [14]	A randomized clinical trial (74 patients diagnosed with plantar fasciitis)	NPRS, FFI	Group A: needling in combination with low tape and a standard treatment protocol. Group B: standard treatment protocol with low dye tapes	Individuals in group A noticed a substantial reduction in functional impairment and pain	Low-dye tape, combined with dry needling and conventional physiotherapy, proved beneficial in treating the painful symptoms
Yadav et al. 2021 [15]	A quasi-experimental study (30 individuals who were suffering from plantar fasciitis)	VAS, WBLT	Group A: stretching of calf and plantar fascia. Group B: foam rolling and self-stretching	All outcome measures (VAS, WBLT) had statistical significance in both groups (p < 0.001)	Foam roller was more successful and significantly improved ROM relative to simple stretching
Kashif et al. 2021 [16]	Single-blind randomized controlled trial. (60 patients with plantar fasciitis were enrolled in the study)	VAS, FADI	Group A received rigid tapping, 15 minutes of stretching, and subtalar mobilization. Group B received conventional physiotherapy	Functional impairment improved more in group A than in group B (p < 0.05).	In comparison to conventional treatment, subtalar mobilization with movement was found to be more effective
Pearce et al. 2021 [17]	Correlation analysis (33 patients with a history of heel pain and diagnosed as plantar fasciitis were included)	Gastrocnemius tightness (measured by goniometer), VAS	Stretching of gastrocnemius, use of socks or a night splint, as well as silicone heel insoles	The mean gastrocnemius tightness was 22 degrees at the beginning and 9 degrees after the end of the therapeutic intervention	A statistically significant association was observed in this study, which is the first to measure the relationship between gastrocnemius tightness and the severity of heel pain in plantar fasciitis
Ranbhor et al. 2020 [18]	A randomized controlled trial (50 participants with plantar fasciitis)	VAS, PPT, WBLT	Intervention group: foam rolling for the plantar fascia and calf muscles. Traditional group: calf muscle and plantar fasciitis self-stretches	Outcome indicators revealed a statistically significant difference (p < 0.001) when analyzed within groups	Stretching and foam rolling treatments both appeared to aid with pain management and ROM
Cohena-Jimenez et al. 2020 [19]	A randomized controlled trial (76 people completed the treatments in a methodical manner)	VAS, Roles and Maudsley score	Group A: foot orthoses. Group B: placebo insoles	The custom-designed foot splint was rated good and excellent in the medium-to-long term	Patients benefit from wearing a personalized foot orthosis because it reduced foot discomfort and enhanced foot functioning
Kumar et al. 2020 [20]	An experimental study (30 subjects who met all the requirements were selected)	VAS	Group A: calcaneal taping. Group B: conventional therapy	The outcome indicated that the experimental group experienced minimal discomfort than the control group	In both groups, the study revealed a considerable shift when comparing post-test results to pre-test results
Naruseviciute and Kubilius 2020 [21]	A randomized participant-blind controlled trial (102 individuals with plantar fasciitis)	VAS, Pressure algometry, Sonography.	Group 1: high-intensity laser therapy. Group 2: LLLT	Results across the groups did not differ significantly. Participants’ opinions in favor of the HILT group varied between the groups in a statistically significant manner	Between groups, there was no statistically significant difference
Kaur and Koley 2020 [22]	A randomized controlled trial (50 individuals who met the inclusion criteria)	VAS, FFI.	Group A: calf stretching. Group B: Achilles tendon stretching	More definite improvements were seen in patients who received Achilles tendon stretching	Stretching the Achilles tendon for four weeks doubled the number of patients reporting meaningful alleviation from symptoms than calf stretching
Bagcier and Yilmaz 2020 [23]	A quantitative prospective randomized clinical trial (40 patients were diagnosed as plantar fasciitis)	VAS, PPT, FFI, gait parameters	Group 1: cold application - ESWT. Group 2: cold application - ESWT-dry needling	Statistically significant ( p< 0.005) were seen in both groups	This study sought to add to the body of knowledge by combining ESWT with dry needling for the first time in the intervention. The two therapy methods are equally affordable and simple to use
Bahar-Ozdemir and Atan 2020 [24]	A randomized controlled trial (45 patients who met the inclusion criteria)	VAS, HTI, FFI	Group 1: ESWT plus low-dye kineso-tapping (KT). Group 2: ESWT plus, sham-taping. Group 3: ESWT	There were no differences between the three groups regarding VAS and HTI alterations	Even though cumulative sham-taping and ESWT alone and low-dye KT with ESWT were superior for improving foot function, they did not significantly reduce pain or heel tenderness due to plantar fasciitis
Gupta et al. 2020 [25]	A double-blind randomized controlled trial (140 individuals with plantar fasciitis were randomized into four groups)	FFI, FADI	Group 1: standard medical care (patients with analgesics). Group 2: heel pads made of silicone and moist heat treatment. Group 3: active plantar fascia stretching with sham calf stretching. Group 4: active calf muscle stretching with sham plantar fascia stretch	In all four groups, FFI and FADI exhibited statistically significant improvement at 12 months	Plantar fascia stretching led to a substantially enhanced efficiency in the outcome measure
Narin et al (2020) [26]	A randomized controlled trial (41 individuals with plantar fasciitis)	Outcome measure: VAS, AOFAS	Group 1: radial ESWT at 15 Hz. Group 2: radial ESWT at 10 Hz	Following rESWT, mean VAS scores decreased. Significant improvements in mean AOFAS scores from pre-intervention to post-intervention were seen in both the groups	There were no statistically significant changes between two groups that received rESWT at two different frequencies. However, the lower frequency group improved faster
Naik and Singh (2019) [27]	An experimental study (17 individuals with plantar fasciitis)	Algometer to measure pain, non-contact IRT, FAAM scale	Matrix therapy for one session	All outcomes were statistically significant (p < 0.005) before and after the intervention	Patients who received the therapy reported reduced pain, increased skin temperature, and enhanced functional abilities
Tong-on et al. (2019) [28]	A randomized controlled trial (84 patients were diagnosed as plantar fasciitis)	VAS, gait parameters	Strengthening exercise: toe flexor exercise/ankle invertor exercise/ankle evertor exercise/high load training. Stretching exercise: gastrocnemius muscle/soleus muscle/plantar fascia	Considering intragroup analyses, pain and gait parameters in both groups were all significantly different. No significant changes in any of the gait parameters were found for the intergroup comparisons	Programs of stretching and strengthening exercises both dramatically decreased pain and enhanced gait in patients
Riel et al. (2019) [29]	A randomized trial (70 participants who follows inclusion criteria)	FHSQ	The experimental group included exercises for heel raises, patient education, and a silicone heel cup. Control group: heavy-slow resistance training for the heel lift exercise, silicone heel cups, and patient education	After 12 weeks, there was no substantial difference in the questionnaire score between the groups	Over the course of 12 weeks, self-dosed and pre-planned heavy-slow resistance training regimens had equal impacts on outcomes. Most persons with plantar fasciopathy cannot get their symptoms under control with these regimens alone
Okur and Aydin 2019 [30]	Prospective randomized controlled study (83 patients were included in the study)	VAS, FFI, FHSQ	Group I: ESWT. Group II: CFO	In terms of pretreatment demographics and evaluation criteria, there was no statistical distinction among the groups	Both interventions are completely interchangeable

Discussion

The alignment of plantar fascia plays an essential role in the maintenance of the arch during locomotion. Inefficient foot function results in impaired foot motion at different gait cycle phases. Excessive stress irritates both the plantar fascia and the calcaneal tubercle. Excessive pronation might result in tibialis posterior weakness and plantar fascia lengthening. Because of the imbalance during the propelling phase of ambulation, the elongation inhibits the optimal utilization of the foot’s windlass mechanism. This function can be restored by strengthening the tibialis posterior and intrinsic muscles. Plantar fasciitis in the pes cavus is characterized by the failure of the foot to distribute equal force. An excessive amount of stress is exerted on the muscles and joints of the lower limbs due to poor foot posture, which can cause anatomical changes across the body. If this excessive tension is left untreated, it results in persistent musculoskeletal discomfort. An overpronated foot position is characterized by a misaligned foot. When the dome of the foot is repeatedly exposed to impact loading, flattening outward and diminishing stability in the region around the ankle joint. As a result of this instability and soreness, increased stress is transmitted via the knees, which display diminished joint space.

This posture further contributes to pelvic misalignment. Core strength is necessary to align the pelvis with the spine, allowing for appropriate femoral rotation. A lack of core muscle strength indicates that keeping the pelvis in the correct position for pain-free movement over time becomes challenging. Both feet bear the body’s load, and ailments of the feet in the plantar fasciitis impact the lower back by altering posture or gait. Internal rotation of the entire lower limb and pelvic tilt is caused by overpronation, defined as the excessive inward foot rolling toward the arches during walking. As a result, the spine’s alignment gets distorted, compromising balance and putting more strain on all soft tissue structures related to their bone attachments. A significant predictor of performance in any test of balance and functional ability, pain can compromise dynamic balance control during load-bearing activities.

The exercise treatment approach provides significant health benefits. It seeks to fortify damaged tissues. Heel discomfort may be effectively treated by performing moderate, repeated exercise to strengthen the foot’s soft tissues. Slow strengthening exercises may promote tissue healing, stretching of the soleus, gastrocnemius, and Achilles resulting in lower adaptive risk factors for plantar fasciitis, which may be caused by recurrent microtrauma [20]. The study by Pearce et al. reported a substantial statistically significant correlation between gastrocnemius tightening and the extent of foot pain in plantar fasciitis. The authors suggested that exercises to stretch the gastrocnemius should be the primary management [17]. Yadav et al. assessed the effects of self-stretching alone versus self-stretching plus foam rolling on pain and range of motion in individuals with plantar fasciitis. The study demonstrated that patients with plantar fasciitis can alleviate pain and enhance joint mobility and range by self-stretching and foam rolling [15].

In this review, the available information was compiled systematically, and the risk of bias in pertinent research and trials was evaluated. Based on the design and including a population of interest, 20 studies were homogeneous. This makes objective comparison possible. The combination of these elements strengthens the study’s findings based on the best data available. However, it is crucial to consider the inherent limitations of the data presented in this article. More than 20 studies out of 189 may be needed to draw firm conclusions. More clinical studies with larger sample sizes are required to assess the findings thoroughly.

Study limitations

It is challenging to exclude the possibility of selection bias in this review. Because this assessment only considered works published in the English language, linguistic bias cannot be disregarded. Despite our best efforts, the restricted availability of research articles from databases and publications prevented us from finding all pertinent studies. Additionally, there needs to be more information on the cost-effectiveness of different plantar fasciitis treatment options. Lastly, methodological flaws, such as small sample sizes, further hampered the review’s meager amount of data.

## Conclusions

An analysis of the trials considered in accordance with the inclusion and exclusion criteria revealed that therapeutic interventions, to varying degrees, alleviate pain, boost foot function, and enhance the patient’s well-being. More than 90% of patients experience successful results with Achilles tendon, plantar fascia, and foam roller stretching than the manual calf and hamstring stretching. Modern methods such as shockwave therapy, laser therapy, taping, dry needling, gel heel cushion, arch support, and night splint are more efficient than traditional methods. Shockwave treatment has better long-term significant results and no significant results compared to different frequencies. Movement with mobilization for the subtalar joint shows increased joint mobility and reduced pain. A combination of dry needling with taping results in considerable pain reduction. High-intensity lasers are more beneficial than lower-intensity lasers. The combination of extracorporeal shock wave therapy with dry needling has remarkable results in the outcome measures. The combination of advanced interventions and standard traditional treatments more significantly impacts the treatment outcome.
